# HIV protease inhibitors restore amphotericin B activity against *Candida*

**DOI:** 10.1371/journal.pone.0324080

**Published:** 2025-05-29

**Authors:** Yehia Elgammal, Ehab A. Salama, Mohamed N. Seleem

**Affiliations:** 1 Department of Biomedical Sciences and Pathobiology, Virginia-Maryland College of Veterinary Medicine, Virginia Polytechnic Institute and State University, Blacksburg, Virginia, United States of America; 2 Center for One Health Research, Virginia Polytechnic Institute and State University, Blacksburg, Virginia, United States of America; Cleveland Clinic Lerner Research Institute, UNITED STATES OF AMERICA

## Abstract

*Candida auris* is an invasive fungal pathogen, representing a global public health threat. It is characterized by high mortality rates among infected individuals, significant antifungal resistance, and a remarkable ability to persist in healthcare environments. While amphotericin B is one of the most powerful antifungal agents for treating *Candida* infections, approximately 30% of *C. auris* isolates demonstrate resistance to it. Thus, the development of novel antifungal therapies is vital for tackling its life-threatening infections. In this study, we identified four HIV protease inhibitors (atazanavir, saquinavir, lopinavir and ritonavir) as strong potentiators of amphotericin B against *C. auris*. A synergistic effect between HIV protease inhibitors and amphotericin B was observed against 15 *C. auris* isolates with fractional inhibitory concentration index (FICI) ranging from 0.09 to 0.50. Additionally, the combinations between HIV protease inhibitors and amphotericin B showed fungicidal effect, significantly reducing the viable cell count in the time-kill assay within 6 hours. Furthermore, the combinations inhibited biofilm formation of *C. auris* by 60–75% and exhibited a remarkable suppression of *C. albicans* hyphae. The *in vivo* treatment with HIV protease inhibitors combined with amphotericin B resulted in a significant reduction of *C. auris* colony-forming units (CFU) by 1.7–2.6 Log_10_ in the *C. elegans* model. These findings suggest that HIV protease inhibitors, in combination with amphotericin B, are promising candidates for the development of novel antifungal drugs to treat *Candida* infections.

## Introduction

Invasive fungal infections pose a significant global health challenge, contributing to over 1.5 million deaths annually [1]*.* Among these pathogens, *Candida auris* (*C. auris*) stands out as a multidrug-resistant pathogenic fungus responsible for severe systemic infections, often leading to death, particularly in patients within long-term healthcare facilities [2,3]. Since its discovery in 2009, *C. auris* has rapidly spread across the globe, with confirmed cases in over 45 countries [4,5]. Over the past decade, *C. auris* has been the source of numerous hospital outbreaks, making it a serious public health concern [6]. Additionally, this fungus is difficult to eradicate from healthcare environments due to its ability to survive for prolonged periods on surfaces and its high rates of resistance to antifungals [7]. Unlike other *Candida* species, *C. auris* poses an elevated risk to patients with underlying comorbidities, immunocompromising conditions, or those with invasive medical devices in long-term healthcare settings. These individuals are particularly vulnerable to severe localized and/or systemic infections [2,8]. Consequently, the World Health Organization (WHO) has classified *C. auris* as a critical-priority fungal pathogen due to its high transmission rates, tendency to cause hospital outbreaks and its resistance to multiple antifungal drugs [9,10].

Amphotericin B (AMB) is one of the first discovered antifungals with a potent fungicidal activity. Despite its toxic side effects, it remains the treatment of choice for severe fungal infections [11]. While AMB is highly effective against *Candida spp.*, the emergence of AMB-resistant species, especially *C. auris*, highlights the urgent need for new therapies to treat systemic infections. Therefore, there is a pressing need to develop co-drugs that enhance the antifungal activity of AMB and reduce its toxicity, providing an effective strategy to combat infections caused by these emerging fungal pathogens.

Biofilm and hyphae formation are crucial virulence factors known to significantly contribute to the pathogenesis of *Candida* species [12]*.* Biofilm is known to provide a protective environment that allows the fungus to adhere to surfaces and persist under harsh conditions [13]. The biofilm matrix is highly resistant to both the host immune response and antifungal agents, which complicates treatment efforts and often leads to chronic infections [14]. Infections associated with biofilms, such as those forming on medical devices, are very difficult to treat as biofilms can tolerate antifungals at concentrations higher than those required to inhibit planktonic fungal cells [13]. Due to the significant resistance of fungal biofilms to existing antifungal medications, effective treatment of infections typically necessitates high doses of antifungals along with the removal of the colonized medical devices [15]. Hyphae formation is another key component of *Candida* pathogenesis [16]. Hyphae, typically produced by *C. albicans* but absent in *C. auris*, are elongated, filamentous forms of the fungus that enable *Candida* to penetrate epithelial barriers and facilitate tissue invasion [17,18]. Moreover, hyphal cells contribute to the structural integrity of biofilms, making them more resistant to antifungal treatment and host defenses [19]. By targeting these two major virulence factors, biofilm and hyphae, antifungals can potentially undermine the protective barriers that make *Candida* infections so difficult to treat. New strategies aimed at disrupting biofilm formation, preventing hyphal development, or combining antifungals with other inhibitory agents could offer more effective treatment options for patients suffering from *Candida* infections, particularly those caused by resistant strains like *C. auris* [20–22].

Combination therapy has emerged as a valuable approach to prevent drug resistance and restore antifungal efficacy. It has been successfully used to treat several fungal infections, particularly those caused by multidrug-resistant fungal pathogens [23,24]. Identifying compounds that resensitize infections to existing antifungals could extend the lifespan of current treatments. *In vitro* and *in vivo* studies suggest that combining existing antifungal agents may be effective in combating *C. auris* [25–27].

In this study, we identified that four HIV protease inhibitors (HIV-PIs), atazanavir (ATV), saquinavir (SQV), lopinavir (LPV) and ritonavir (RTV), were able to enhance the antifungal efficacy of AMB against *C. auris*. Our goal is to enhance the efficacy of AMB against *C. auris* while reducing its associated toxicity by using HIV-PIs. Furthermore, we evaluated the efficacy of the combinations of HIV-PIs and AMB in inhibiting *C. auris* biofilm formation and *C. albicans* hyphal growth. Finally, we assessed the *in vivo* efficacy of HIV-PIs/AMB combinations using a *C. elegans* model of *C. auris* infection.

## Materials and methods

### *C. auris* strains, chemicals, and compounds

Fifteen *C. auris* isolates were acquired from the CDC and Biodefense and Emerging Infections (BEI) resources. *C. albicans* SC5314 (Wild-type strain) was provided by American Type Culture Collection (ATCC). Chemicals and drugs were provided from following chemical vendors: yeast peptone dextrose (YPD) broth (Becton, Dickinson and Company, Franklin Lakes, NJ, USA); YPD agar (DOT Scientific Inc, Burton, MI, USA); RPMI 1640 (Gibco, Grand, Island, NY, USA); 3 (N-Morpholino) propane sulfonic acid (MOPS) (Fisher Bioreagents, Fairlawn, NJ, USA); crystal violet (Acros Organics, New Jersey, USA); phosphate-buffered saline (PBS) (Corning, Manassas, VA, USA); AMB (Chem-Impex International, Wood Dale, IL, USA); Nelfinavir (Cayman chemical, Ann Arbor, Michigan, USA); RTV (TCI America, Portland, OR, USA); Amprenavir, darunavir, indinavir, tipranavir, LPV and ATV (Ambeed, Arlington Heights, IL,USA); SQV (Biosynth Carbosynth, San Diego, CA, USA).

### Antifungal susceptibility and checkerboard assay

Drug susceptibility testing is carried out to determine the minimum inhibitory concentrations (MICs) of AMB (8–0.06 µg/mL) and HIV-PIs (128-1 µg/mL) using a broth microdilution protocol modified from the Clinical and Laboratory Standards Institute M-27A methods [28]. Briefly, HIV-PIs at 128 µg/mL and AMB (positive control) at 8 µg/mL were added to the first row of a 96-well plate, followed by twofold serial dilution. DMSO was included as a negative control. Each well contained 100 µL of RPMI medium and a final cell density of 10³ CFU/mL. The plate was then incubated at 35°C for 24 hours. After incubation, the MICs of the drugs were determined visually. The combined effect of AMB with HIV protease inhibitors was evaluated against *C. auris* isolates via checkerboard assay [29–31]. The synergistic interaction of the combination was determined based on the value of the fractional inhibitory concentration index (FICI). FIC values ≤0.5 indicate a synergistic effect, 0.5 < FIC < 2 an additive effect and 1 < FIC < 4 an antagonistic effect [32].

### Time-kill assay

The time-kill curve was determined as previously described [33,34]. The inoculum of *C*. *auris* AR0390 was adjusted to ~1 × 10^4^ CFU/mL in RPMI-1640 medium and treated with HIV-PIs (16 μg/mL), AMB (0.5 μg/mL), or a combination of both. Untreated *C. auris* cells were used as the drug-free control. The suspensions were incubated in 96-well plates at 35°C. Aliquots of 100 μL were withdrawn, at predetermined time points (0, 6, 12, 24, 30, 36 and 48 hours), diluted in PBS and plated on YPD agar and incubated at 35°C for 24 hours for CFU determination.

### Examination of *C. albicans* yeast-to-hyphae inhibition

The impact of HIV-PIs in combination with AMB on hyphae formation of the *C. albicans* SC5314 was assessed as previously described [20]. Cells were exposed to either HIV-PIs (16 µg/mL), AMB (0.06μg/mL, 0.125 × MIC) or a combination of both and diluted in RPMI 1640 medium at a concentration of 1 × 10^5^ CFU/mL. The cultures were incubated at 35 ºC for four hours. Afterwards, media was removed, and *Candida* cells were stained with crystal violet for 30 minutes. Then, cells were washed with PBS. The morphology of the cells and its hyphal development were examined, and images were captured using Olympus BX41 microscope.

### Biofilm inhibition using crystal violet assay

The effect of the HIV-PIs in preventing biofilm formation was evaluated as reported previously [21,35]. Briefly, HIV-PIs (16 μg/ml) and AMB (0.06 μg/ml), diluted in RPMI 1640 medium, were added to 96-well plates containing ~10^6^ CFU/ml of *C. auris* AR0390 cells, and the plates were incubated at 35°C for 24 hours. Adherent biofilms were stained for 30 min with 100 μl of 0.1% (wt/vol) crystal violet. After crystal violet was removed, they were washed 3 times with 200 μl of distilled water and the plates were allowed to dry. The resultant biofilm biomasses were quantified by dissolving the crystal violet-stained biofilms in 100 μl of 70% ethanol before recording absorbance values (OD_600_).

### *Caenorhabditis elegans* (*C. elegans*) infection model

We used the *C. elegans* infection model to examine the HIV-PIs’ ability to enhance the antifungal action of AMB *in vivo* [36,37]. In brief, synchronized worms [strain AU37 genotype (*glp-4(bn2) I; sek-1(km4) X*)] in L4 phase were infected with 1 × 10^6^ CFU/ml *C. auris* AR0390 for 3 hours at room temperature. To eliminate non-ingested yeast cells, infected nematodes were washed with PBS five times. Next, the infected nematodes were treated with DMSO (1.6%), HIV-PIs (LPV, RTV, ATV, or SQV) (16 μg/ml), AMB (0.5 μg/ml), or a combination of both. Worms were treated and incubated at 25 °C for 24 hours before being examined microscopically for vitality. Worms were then vigorously vortexed with silicon carbide beads for at least 15 minutes to release the ingested *C. auris* cells. After serial dilution, the *C. elegans* homogenates were plated onto YPD agar plates containing gentamicin (100 μg/ml). CFU per worm were obtained on YPD agar after 24 hours of incubation at 35°C.

## Results

### Screening of HIV protease inhibitors against *C. auris*

We tested the activity of nine HIV-PIs individually and with sub-inhibitory concentration of AMB at 0.25 × MIC (0.5 μg/ml). The results revealed that a significant reduction in the MICs of several HIV-PIs when combined with AMB, suggesting a synergistic interaction between the HIV-PIs and AMB (**Table 1**). Among the tested HIV-PIs, ATV, SQV, LPV, and RTV are the most potent co-drugs that restore the activity of AMB against *C. auris* with MICs ranging between 0.5–1 μg/mL. These findings underscore the potential of HIV-PIs to restore or enhance the antifungal activity of AMB against drug-resistant *C. auris* strains.

**Table 1:** The minimum inhibitory concentrations (MICs) of nine HIV protease inhibitors alone and with sub-inhibitory concentration of amphotericin B against *C. auris* AR0390.

HIV protease inhibitors	MICs of HIV protease inhibitors (μg/ml)	
	Alone	+ Amphotericin B (0.25 × MIC)
**Lopinavir**	>128	1
**Saquinavir**	>128	0.5
**Atazanavir**	>128	1
**Ritonavir**	>128	0.5
**Amprenavir**	>128	32
**Darunavir**	>128	32
**Indinavir**	>128	8
**Nelfinavir**	>128	>32
**Tipranavir**	>128	>32

### *In vitro* synergistic activity of HIV-PIs against a panel of *C. auris* isolates

The *in vitro* interaction between the most potent HIV-PIs (ATV, SQV, LPV, and RTV) and AMB was investigated against a panel of 15 clinical isolates representing the four main clades of *C. auris*. Among these isolates, 9/15 (60%) were resistant to AMB (MIC = 2 µg/mL). Surprisingly, all four HIV-PIs demonstrated a synergistic interaction with AMB in 100% (15/15) of the *C. auris* isolates with FICI ranging between 0.09 and 0.50 (**Tables 2** and **3**).

**Table 2:** HIV protease inhibitors, atazanavir (ATV) and saquinavir (SQV) restore amphotericin B (AMB) activity against *Candida auris* isolates.

*C. auris* Isolate ID	Clade	AMB/ATV combination				AMB/SQV combination			
		MIC (µg/ml)		ΣFICI	Mode	MIC (µg/ml)		ΣFICI	Mode
		Alone	Combined			Alone	Combined		
AR0381	II	1/ > 128	0.125/4	0.14	SYN	1/ > 128	0.125/4	0.14	SYN
AR0382	I	1/ > 128	0.125/8	0.16	SYN	1/ > 128	0.125/2	0.13	SYN
AR0383	III	1/ > 128	0.125/8	0.16	SYN	1/ > 128	0.125/2	0.13	SYN
AR0384	III	2/ > 128	0.25/8	0.16	SYN	2/ > 128	0.25/4	0.14	SYN
AR0385	IV	2/ > 128	0.5/2	0.26	SYN	2/ > 128	0.5/2	0.26	SYN
AR0386	IV	4/ > 128	0.5/8	0.19	SYN	4/ > 128	0.5/4	0.16	SYN
AR0387	I	1/ > 128	0.06/8	0.13	SYN	1/ > 128	0.06/4	0.09	SYN
AR0388	I	4/ > 128	1/16	0.38	SYN	4/ > 128	1/4	0.28	SYN
AR0389	I	4/ > 128	1/16	0.38	SYN	4/ > 128	1/4	0.28	SYN
AR0390	I	2/ > 128	0.25/8	0.19	SYN	2/ > 128	0.25/4	0.16	SYN
NR-52713	I	2/ > 128	0.5/16	0.31	SYN	2/ > 128	0.5/2	0.26	SYN
NR-52714	I	2/ > 128	0.5/8	0.28	SYN	2/ > 128	0.5/4	0.27	SYN
NR-52715	I	1/ > 128	0.125/8	0.16	SYN	1/ > 128	0.125/2	0.13	SYN
NR-52716	I	1/ > 128	0.25/2	0.26	SYN	1/ > 128	0.125/4	0.14	SYN
NR-52717	I	2/ > 128	0.5/4	0.27	SYN	2/ > 128	0.25/4	0.14	SYN

**Table 3:** HIV protease inhibitors, lopinavir (LPV) and ritonavir (RTV) restore amphotericin B (AMB) activity against *Candida auris* isolates.

*C. auris* Isolate ID	AmB/LPV combination				AmB/RTV combination			
	MIC (µg/ml)		ΣFICI	Mode	MIC (µg/ml)		ΣFICI	Mode
	Alone	Combined			Alone	Combined		
AR0381	1/ > 128	0.25/4	0.27	SYN	1/ > 128	0.25/1	0.25	SYN
AR0382	1/ > 128	0.5/2	0.50	SYN	1/ > 128	0.25/8	0.28	SYN
AR0383	1/ > 128	0.25/8	0.28	SYN	1/ > 128	0.25/16	0.31	SYN
AR0384	2/ > 128	0.5/2	0.26	SYN	2/ > 128	0.5/1	0.25	SYN
AR0385	2/ > 128	0.5/4	0.27	SYN	2/ > 128	0.5/1	0.25	SYN
AR0386	4/ > 128	0.5/4	0.28	SYN	4/ > 128	0.5/2	0.26	SYN
AR0387	1/ > 128	0.06/8	0.13	SYN	1/ > 128	0.5/2	0.26	SYN
AR0388	4/ > 128	1/4	0.28	SYN	4/ > 128	0.25/4	0.27	SYN
AR0389	4/ > 128	1/16	0.38	SYN	4/ > 128	0.25/2	0.26	SYN
AR0390	2/ > 128	0.5/4	0.28	SYN	2/ > 128	0.5/4	0.27	SYN
NR-52713	2/ > 128	0.5/8	0.28	SYN	2/ > 128	0.5/2	0.26	SYN
NR-52714	2/ > 128	0.5/8	0.28	SYN	2/ > 128	0.5/2	0.26	SYN
NR-52715	1/ > 128	0.125/8	0.16	SYN	1/ > 128	0.25/4	0.27	SYN
NR-52716	1/ > 128	0.25/8	0.28	SYN	1/ > 128	0.25/2	0.26	SYN
NR-52717	2/ > 128	0.5/16	0.31	SYN	2/ > 128	0.5/4	0.27	SYN

### The combinations of HIV-PIs and AMB exhibit fungicidal activity in a time-kill assay

**Fig 1:**
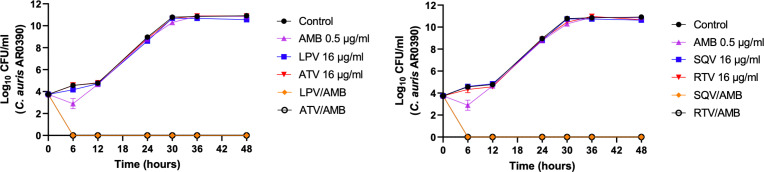
Time-kill assay of *C. auris* strain AR0390 treated with individual HIV protease inhibitors, amphotericin B (AMB), or combinations of both. Fungal load, measured as Log_10_ CFU/ml, was recorded at specific intervals over 48 hours to evaluate the effects of combination treatments on *C. auris* viability. The treatments included combinations of AMB with atazanavir (ATV/AMB), saquinavir (SQV/AMB), lopinavir (LPV/AMB), and ritonavir (RTV/AMB) at concentrations of 16 μg/mL for the HIV protease inhibitors (HIV-PIs) and 0.5 μg/mL for AMB. Statistical analysis, assessed using two-way analysis of variance (ANOVA), revealed a highly significant difference between the HIV-PI/AMB combination treatments and AMB-treated cells alone at 6 hours and beyond, with a *P-value < 0.0001*.

To determine the killing kinetics of the combinations, we conducted a time-kill assay. As shown in **Fig 1**, the HIV-PIs in combination with sub-inhibitory concentrations of AMB (0.5 μg/ml) exhibited fungicidal activities against *C. auris* AR0390 within 6 hours. On the other hand, *C. auris* growth in the presence of either HIV-PIs (16 µg/ml) or AMB (0.5 μg/ml) was similar to that of the untreated control (DMSO), which confirmed that either HIV-PIs or AMB alone possessed no antifungal activity at the tested concentrations.

### HIV-PIs, in combination with AMB, interfere with the hyphal growth of *C. albicans*

**Fig 2:**
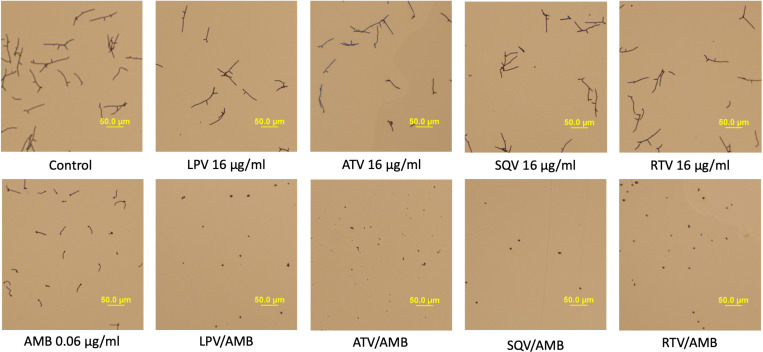
Phase-contrast images of yeast and hyphal forms of *C. albicans* SC5314 after treatment with either of the HIV-protease inhibitors, amphotericin B or a combination of both. Inhibition of *C. albicans* SC5314 hyphal morphology was observed when treated with either of the HIV-protease inhibitors (lopinavir (LPV), atazanavir (ATV), saquinavir (SQV) or ritonavir (RTV)) (16 μg/mL), amphotericin B (AMB) (0.06 μg/mL) or their combinations (LPV/AMB, ATV/AMB, SQV/AMB, RTV/AMB). *C. albicans* cells were incubated at 35°C. The photos were taken 4 hours after treatment.

Since hyphal growth is a key virulence factor during the *C. albicans* infection process, we tested the impact of HIV-PIs on hyphal growth *in vitro*. As shown in **Fig 2**, hyphal formation of *C. albicans* SC5314 was inhibited when cells were treated with the combination of HIV-PIs/AMB at sub-inhibitory concentrations within 4 hours, compared to each drug alone or the untreated control.

### HIV-PIs, in combination with AMB, inhibit the biofilm formation of *C. auris*

**Fig 3:**
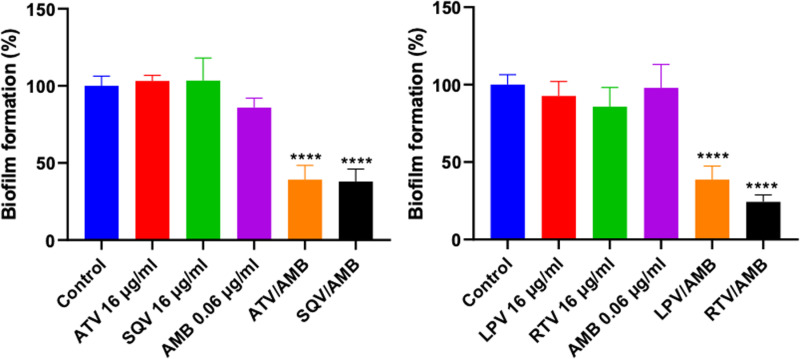
The anti-biofilm activity of HIV-protease inhibitors/amphotericin B (AMB) against *C. auris.* The ability of the HIV-protease inhibitors (atazanavir (ATV), saquinavir (SQV), ritonavir (RTV), lopinavir (LPV) at (16 µg/mL))-amphotericin B (AMB) (0.06 µg/mL) combination to inhibit the formation of *C. auris* AR0390 biofilms was determined by crystal violet assay. **** denote a statistically significant difference of *C. auris* cell treated with the combinations (ATV/AMB, SQV/AMB, LPV/AMB, and RTV/AMB) as compared to the control-untreated cells (*P* < 0.0001).

Since the combinations of HIV-PIs/AMB demonstrated the ability to interfere with hyphae formation, we hypothesized that they would also inhibit biofilm formation. *C. auris* AR0390 was incubated with sub-inhibitory concentrations of HIV-PIs and AMB, and the biofilm mass was stained with crystal violet. As expected, the combinations significantly reduced *C. auris* biofilm formation by 60–75% (**Fig 3**).

### The combinations of HIV-PIs/AMB reduce the fungal burden of *C. auris* in *C. elegans*

**Fig 4:**
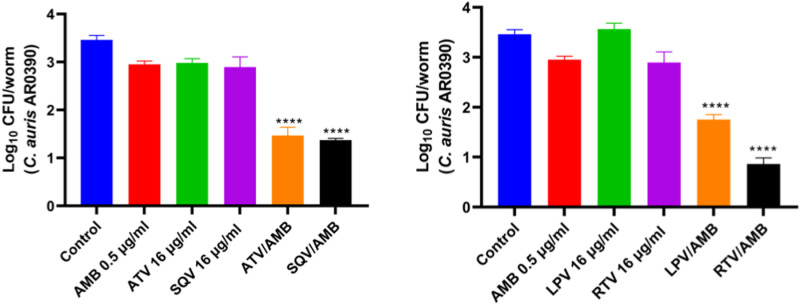
The *in vivo* therapeutic impact of HIV protease inhibitors (HIV-PIs) in combination with amphotericin B (AMB) on *C. auris* infection in the *Caenorhabditis elegans* model. The fungal burden in infected *C. elegans* was quantified by plating homogenized worms on YPD agar and counting colony-forming units (CFUs). The combination treatment groups exhibited a marked reduction in fungal burden compared to the single-drug treatments and control. Data are presented as mean ± SEM, with **** represents *P* value < 0.0001 calculated using one-way ANOVA for Log_10_ CFU.

The promising antifungal activity of the HIV-PIs/AMB *in vitro* and its potent anti-virulence activity against hyphae and biofilm formation encouraged us to evaluate the efficacy of the combinations *in vivo* using *C. elegans* model. To evaluate the *in vivo* antifungal efficacy of the combinations, *C. elegans* were infected with an AMB-resistant strain of *C. auris* AR0390. After 4 hours infection period, the worms were washed and subsequently treated with HIV-PIs (16 μg/ml), AMB (0.5 μg/ml) or a combination of both. The combinations of ATV/AMB, SQV/AMB, LPV/AMB, and RTV/AMB significantly reduced the *C. auris* burden in *C. elegans* by 1.7–2.6 log_10_ (**Fig 4**).

## Discussion

Invasive candidiasis is the most common fungal infection in humans and pose a significant risk to people with weakened immune systems [38]. In contrast to bacterial infections, only three major classes of antifungal drugs—azoles, polyenes, and echinocandins—are available for treating invasive fungal infections. This limitation arises primarily from the eukaryotic nature of fungal cells, which makes it difficult to identify drug targets that are unique to fungi and not shared with human cells [38,39]. Additionally, there are limited new antifungal classes in development, with ibrexafungerp, a triterpenoid, being the first novel class introduced in nearly 30 years [40]. While it may be useful for treating invasive candidiasis, including *C. auris* infections, its clinical use is still limited, and it is currently approved only for the treatment of vulvovaginal candidiasis [5].

AMB is the current standard treatment for fluconazole-resistant fungal infections, as it binds to ergosterol and disrupts the fungal plasma membrane. Despite its toxic potential, it remains useful in the treatment of invasive fungal diseases owing to its broad spectrum of activity, low resistance rate, and excellent clinical and pharmacological outcomes [11]. However*,* 30% of *C. auris* isolates are resistant to AMB, potentially due to the overexpression of several mutated *ERG* genes that lead to reduced ergosterol levels. Moreover, pan-resistant *C. auris* isolates have been reported in the United States [41]. Thus, there is an urgent need to develop co-drugs that enhance the antifungal activity of AMB while reducing its toxicity.

Our group have previously identified HIV-PIs as co-drugs that enhance the activity of azole antifungals (fluconazole, itraconazole and posaconazole) by inhibiting the efflux pump machinery [42–44]. In this study, we identified four HIV-PIs, as potentiators of the antifungal activity of AMB against *C. auris*. All tested HIV-PIs didn’t show any noticeable antifungal activities when tested alone against *C. auris* (MIC > 128 μg/ml). However, four HIV-PIs, namely ATV, SQV, LPV, and RTV, displayed potent synergistic interactions with AMB and restored its antifungal activity against all AMB-resistant *C. auris* isolates. Moreover,  they restored the fungicidal activity of AMB, resulting in eradicating *C. auris* cells within 6 hours.

*Candida* species employs several virulence mechanisms, including biofilm formation, which protects the yeast from environmental stress and contributes to its survival in hostile conditions [45–47] This process is reinforced by hyphal development, aiding in the invasion of host tissues [12]. Both hyphae and biofilm formation are regulated by various genes and transcription factors, and disruptions in these transcription factors markedly reduce the virulence of *Candida* in mouse models of systemic infection [48–50]. For instance, knocking out these transcription factors prolongs the survival of mice infected with *C. albicans* and leads to a marked reduction fungal load within the kidneys, liver and spleen [50]. Moreover, *Candida* virulence factors, such as biofilm and hyphae formation, are not only critical for tissue invasion but also contribute significantly to drug resistance, leading to poor treatment outcomes. These mechanisms collectively enable the fungus to persist in challenging environments and resist antifungal treatments, highlighting the difficulty of managing infections caused by this pathogen. Our group previously demonstrated that HIV-PIs, in combination with posaconazole, significantly inhibited the biofilm formation of *C. auris* [44]. In this study, HIV-PIs, when used in combination with AMB, have been shown to significantly disrupt the hyphal growth of *C. albicans*, and inhibits biofilm formation of *C. auris* by 60–75%. This disruption of the morphological transition limits the ability of *C. albicans* to establish infections and reduces its pathogenic potential. These findings suggest that HIV-PIs, beyond their antiviral properties, could serve as valuable co-drugs to existing antifungal therapies by weakening either *C. albicans* or *C. auris* virulence through inhibition of hyphal growth and biofilm formation, respectively.

A key milestone in drug development is testing the *in vivo* efficacy of the HIV-PIs/AMB combinations in the *C. elegans* model, which highlights its promising therapeutic potential. Notably, the HIV-PIs/AMB combinations resulted in a significant reduction of the *C. auris* burden in *C. elegans*, generating 1.7–2.6 log_10_ reduction. Further evaluation in higher animal models and comprehensive clinical trials are essential to fully establish the potential of the HIV-PIs/AMB combination as a novel therapeutic approach. These promising combinations have demonstrated significant antifungal activity against multidrug-resistant *C. auris*, but their clinical applicability requires further investigation.
